# A proteomics and redox proteomics approach to understanding ARDS heterogeneity

**DOI:** 10.1038/s41598-026-35606-2

**Published:** 2026-01-23

**Authors:** Thomas E. Forshaw, Kirtikar Shukla, Hanzhi Wu, Susan Sergeant, Jingyun Lee, Allen W. Tsang, Peter E. Morris, Kevin W. Gibbs, D. Clark Files, Cristina M. Furdui

**Affiliations:** 1 Department of Internal Medicine, Section on Molecular Medicine, Wake Forest University School of Medicine, Winston-Salem, NC 27157 USA; 2 Department of Biochemistry, Wake Forest University School of Medicine, Winston- Salem, NC USA; 3 Department of Internal Medicine, Section of Pulmonary, Critical Care, Allergy, and Immunologic Diseases, Wake Forest University School of Medicine, Winston-Salem, NC USA; 4 Present Address: Department of Chemistry, North Carolina Agricultural and Technical State University, Greensboro, NC USA; 5 Present Address: PPD, Middleton, WI USA; 6 Present Address: Department of Medicine, Division of Pulmonary/Allergy/Critical Care, University of Alabama at Birmingham, Birmingham, AL USA

**Keywords:** Biomarkers, Diseases, Immunology, Medical research

## Abstract

Acute Respiratory Distress Syndrome (ARDS) is a severe and heterogeneous critical illness characterized by systemic inflammation, lung injury, and profound hypoxemia. To investigate the temporal evolution of molecular features underlying ARDS heterogeneity, we applied advanced proteomics and redox proteomics to matched plasma and bronchoalveolar lavage (BAL) fluid samples collected longitudinally from 16 intensive care unit (ICU) ARDS patients during hospitalization. Exploratory, data-driven hierarchical clustering (Ward method) identified three distinct molecular patterns across patients represented as Groups A, B, and C. This framework was associated with illness severity at study enrollment (Group A profiling patients with more severe illness at enrollment), demonstrated temporal stability across sampling timepoints, and revealed molecular features associated with clinical improvement during hospitalization. Key pathways distinguishing the molecular patterns and consistent with prior findings included the production and detoxification of reactive oxygen species (ROS), Liver X receptor–Retinoid X receptor (LXR/RXR) activation and 24-dehydrocholesterol reductase (DHCR24) signaling, interleukin-12 (IL-12) signaling and production in macrophages, and neutrophil degranulation. Although plasma proteomic profiles were generally consistent with findings in BAL fluid, BAL fluid data were more mechanistically informative and enabled clearer and more consistent interrogation of ARDS molecular heterogeneity. The results highlight the potential value of lung–focused, temporal studies to improve patient stratification and guide future therapeutic strategies. However, the modest cohort size and exploratory nature of this study necessitate cautious interpretation of pathway-level inferences. Future longitudinal studies in larger, independent ARDS cohorts will be required to validate these molecular groups and assess their clinical relevance.

**Supplementary Information:**

The online version contains supplementary material available at 10.1038/s41598-026-35606-2.

## Introduction

Acute Respiratory Distress Syndrome (ARDS) is a severe critical illness characterized by bilateral pulmonary infiltrates, profound hypoxemia, and often necessitates mechanical ventilation for life support^1–3^. This heterogeneous clinical syndrome can arise from direct lung injury (e.g., chest trauma, pneumonia) or indirect lung injury (e.g., extrapulmonary sepsis, pancreatitis).

Histopathologically, ARDS results in increased lung-blood barrier permeability due to epithelial cell injury, inflammatory cell infiltration, interstitial and alveolar edema, capillary thrombosis, and, in some cases, lung fibrosis develops^1–3^. Despite advances in supportive care and extensive research efforts, ARDS-associated mortality remains high at 20–50%, and no targeted pharmacologic therapies have been successfully developed to date^4,5^. The failure of pharmacologic therapies to effectively treat ARDS underscores the clinical and pathological complexity of the syndrome. Therefore, identifying distinct molecularly characterized subphenotypes could pave the way for the development of urgently needed targeted therapeutic strategies^6^.

We hypothesized that molecular patterns in ARDS are dynamic over the disease course, reflecting either clinical improvement or deterioration. To test this hypothesis, we conducted a small exploratory study integrating proteomic and redox proteomic profiling of bronchoalveolar lavage (BAL) fluid and plasma collected longitudinally from 16 critically ill patients with ARDS. Given the well-established links between inflammation, reactive oxygen species (ROS), and protein oxidation^7–10^, we anticipated that redox proteomics would provide mechanistic insights beyond conventional proteomic analysis.

While plasma proteomic profiles generally paralleled those observed in BAL fluid, analyses of BAL fluid yielded deeper mechanistic insight and enabled a more robust interrogation of lung-specific molecular heterogeneity. As expected, inter-patient molecular heterogeneity exceeded longitudinal variability within individual patients, with only limited exceptions. Notably, redox proteomic analyses revealed that higher levels of antioxidant proteins were associated with lower ARDS severity, supporting a functional link between redox regulation and disease burden.

Collectively, these findings provide proof-of-concept for the application of integrated proteomic and redox proteomic analyses to patient-derived clinical samples to improve characterization of ARDS molecular heterogeneity and to identify mechanistically relevant pathways that may inform therapeutic targeting. Although exploratory, this work lays the foundation for larger, prospective studies and underscores the importance of increased sampling frequency during the early, critical hours following ICU admission to more fully capture disease dynamics and treatment responses.

## Results

In total, 16 critically ill ARDS patients were enrolled in the study from August 6, 2014 until November 20th 2015. Of those, 15 underwent bronchoscopy on study admission (SA, day 0); 10 patients underwent a second bronchoscopy on day 2, and of these patients, 7 underwent a third bronchoscopy procedure on day 4 or 6. One patient underwent bronchoscopy only on days 2 and 4. Day 0 blood samples were not obtained for two of the patients (patients 4 and 5).

Baseline demographic and clinical data of enrolled patients are reported in Table 1. The participant population was 31% female, the age range was 31 to 83 (median 66.5 years), and all were white, non-Hispanic individuals. Four patients (25%) had mild ARDS at enrollment, 6 patients had moderate ARDS at enrollment, and 6 patients had severe ARDS at enrollment. Clinical data included SOFA and APACHE scores, SpO_2_/FiO_2_ ratio, and lactate levels. Of the enrolled patients, two (patients 1 and 2) died while in the ICU, and two other participants (patients 7 and 16) died between the time of hospital discharge and day 90.

**Table 1:** Study demographics and clinical data. Enrolled patients underwent medical care while in the ICU during their hospital stay. All patients were white, non-Hispanic individuals. Clinical data and ARDS scoring values were collected at admission (SA, study admission/Day 0).

Patient ID	Age	Sex	APACHE	SOFA	Vent free days	ICU LOS	Hospital LOS	Lactic acid	SA SpO_2_/FiO_2_
1	46	M	31	10	0	5	5	2.1	250
2	32	M	24	10	0	8	8	5.8	250
3	64	F	27	11	17	7	25	1.3	228
4	60	F	36	12	18	12	21	2.9	155
5	42	M	25	9	16	23	40	2.4	100
6	79	M	16	3	24	7	13	0	152
7	31	M	16	5	25	4	6	-	192
8	74	M	23	5	14	17	31	2.1	233
9	77	M	26	11	26	3	6	2.9	250
10	69	F	35	9	19	9	21	0.8	182
11	83	M	36	11	22	8	23	-	233
12	76	M	27	11	20	14	17	1	184
13	61	M	22	9	28	5	34	4.6	164
14	51	F	34	13	21	11	18	1.1	238
15	76	F	18	12	26	3	8	2.5	233
16	71	M	24	7	15	13	19	-	238

### Patient stratification based on proteomic and redox proteomic profiles

Mass spectrometry-based proteomic and redox proteomic data were collected for the BAL fluid and plasma specimens obtained from the study cohort as early as practically possible after diagnosis [BAL fluid (Day 0, *n* = 15; Day 2, *n* = 1); plasma (Day 0, *n* = 14; Day 2, *n* = 2)]. The analysis identified a higher number of proteins in the BAL fluid compared to plasma specimens (773 vs. 295 proteins; and 334 vs. 184 reversibly oxidized proteins). The Venn diagrams with the results compared by analysis (proteomics vs. redox proteomics in BAL fluid and plasma) and by specimen (plasma vs. BAL fluid proteomics and redox proteomics) are presented in Supplemental Figure S1.

**Fig. 1:**
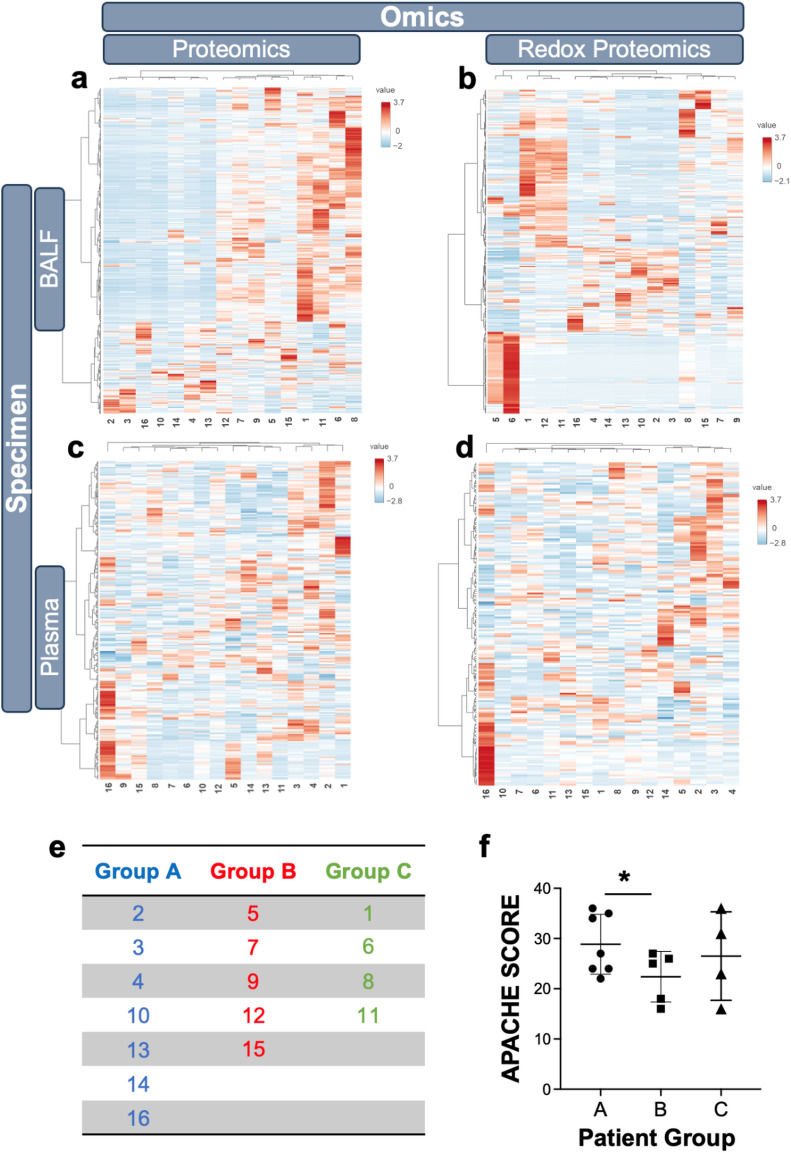
Heatmap analysis clustering of patients by levels of each identified protein. Biospecimen proteomic signatures were statistically analyzed by Ward clustering and Euclidean distancing. Using heatmap analysis results of BAL fluid (**a**,** b**) and plasma (**c**,** d**) proteomic (**a**,** c**) and redox proteomic (**b**,** d**) data, patterns of similarly grouped patients were identified (**e**). X-axis shows the deidentified patient number and value represents autoscaled protein abundance (Z score). The APACHE score (**f**) showed a statistically significant difference between groups A and B when compared by T-test (1-tail; *p* = 0.0418). The data are the mean ± SD.

To prevent data bias, we applied unsupervised blind hierarchical heatmap analysis with Ward clustering algorithms to all four datasets derived from samples (BAL fluid and plasma; proteomics and redox proteomics) obtained early after diagnosis (Day 0 or 2 as described above). Notably, the BAL fluid proteomic and redox proteomic data (Fig. 1a, b) reflecting the lung tissue as site of injury exhibited clearer and consistent pattern of patient grouping compared to plasma data (Fig. 1c, d).

To contextualize these observations, clustering structure was evaluated across all datasets using internal clustering tools (average silhouette width; Supplemental Fig. S2a). Based on this analysis, BAL fluid proteomics was used as the primary data for downstream interrogation, and a three group framework (Fig. 1e) was explored as a hypothesis generating approach to examine heterogeneity rather than as a definite estimate of discrete molecular subphenotypes. For the given number of groups, patient grouping was assigned algorithmically by partitioning the hierarchical dendrogram. BAL fluid proteomics dendrogram (Supplemental Fig. S2b) was largely consistent with BAL fluid redox proteomics dendrogram (Supplemental Fig. S2c), while the fidelity of the plasma data clustering tended to be lower (Supplemental Fig. S2d,e).

Most of the routine clinical parameters (age, SpO2/FiO2 ratio at admission, ventilator-free days, hospital length of stay, and ICU length of stay, lactic acid, SOFA; Supplemental Fig. S3a-g) did not demonstrate a correlation with the BAL fluid proteomic-informed patient groupings. An exception was only a modest difference in the APACHE scores between Groups A and B (Fig. 1f). This suggests that the typical clinical endpoints do not capture molecular underpinnings of ARDS. Importantly, the Lactate Dehydrogenase levels (Supplemental Fig. S3h-i) increased significantly from Group A to B and C in opposite directions to lactic acid as expected.

### Temporal stability of ARDS molecular profiles during ICU hospitalization

**Fig. 2:**
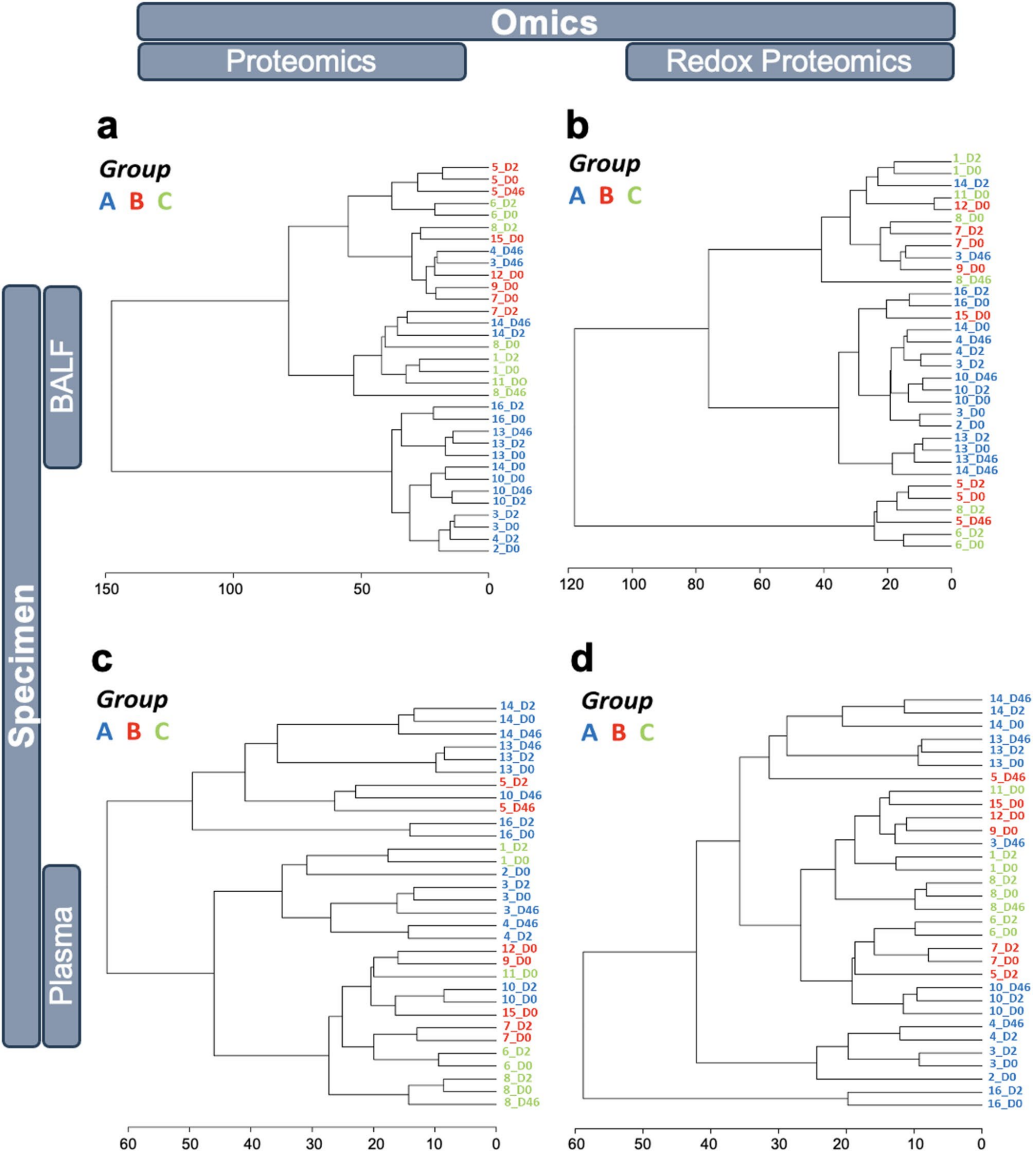
Temporal stability of proteomic and redox proteomic profiles. Proteomic signatures were statistically analyzed by Ward clustering and Euclidean distancing for biospecimens obtained over the time course of the patients’ hospital stay. BAL fluid (**a**,** b**) and plasma (**c**,** d**) proteomic (**a**,** c**) and redox proteomic (**b**,** d**) data are shown using as reference the BAL fluid proteomic-informed patient groupings: Groups A (blue), B (red) and C (green) labels. The biospecimens collection times were at admission (D0), 2 days-post admission (D2), and 4 and/or 6 days-post admission combined (D4-6). The labels represent the patient number followed by biospecimen collection time. X-axis represents clustering distance.

The complex pathology underlying ARDS evolves over the course of disease progression. To investigate whether sampling time post-admission could provide mechanistic insights, analyses were conducted to compare the Day 0 BAL fluid and plasma proteomic and redox proteomic data with Day 2 and Day 4–6 (data for the specimens collected on days 4 and 6 were combined to balance the number of patients across time points). Hierarchical clustering of timecourse proteomic and redox proteomic data (Fig. 2) revealed that the BAL fluid-informed patient groupings defined in Fig. 1e remained largely stable for up to 6 days post-admission as reflected in both the BAL fluid and plasma data. Only a modest time-dependent divergence was observed in the BAL fluid data, with 85% of proteomic (Fig. 2a) and 82% of redox proteomic time point data (Fig. 2b) clustering adjacently by patient. Compared to BAL fluid-derived data, plasma proteomic and redox proteomic data exhibited an even greater temporal stability within individual patients, with 97% and 94% of proteomic and redox proteomic data, respectively, clustering adjacently across time points. Despite this stability, the plasma data demonstrated a more complex hierarchical organization (Fig. 2c, d and Supplemental Fig. S2d, e), thus making it more difficult to discern patient groupings. When applying the BAL fluid proteomics Groups A, B, and C to plasma-derived data, some patients assigned to Group A based on admission (Day 0) profile showed a transition to molecular profiles aligned more with those in Groups B and C (e.g., patient 14 transitioning to Group C at day 2 and Day 4–6; patients 3 and 4 aligning with Group B at Day 4–6) suggesting perhaps an improvement in their condition.

### Mechanistic insights gained from the analysis of BAL fluid data

**Fig. 3:**
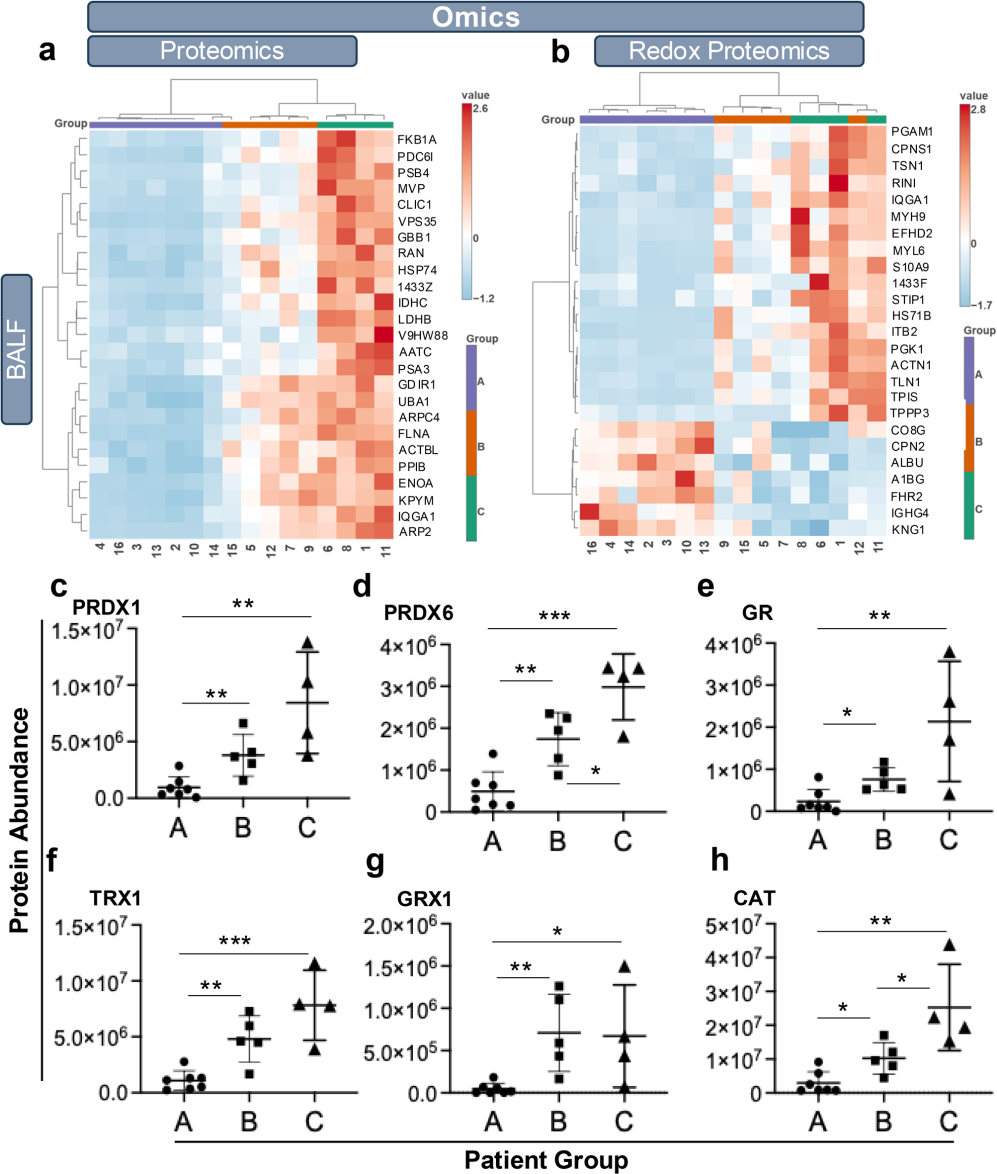
Analyses of key significant proteins in patient groups. BAL fluid proteomic signatures were statistically analyzed by Ward clustering and Euclidean distancing. The top 25 proteins (based on ANOVA, with Fisher’s LSD post-hoc analysis with cutoffs for p-value of 0.05 and FDR at 10%) are shown for BAL fluid proteomic (**a**) and redox proteomic (**b**) data. Value represents autoscaled abundance (Z score) and x-axis shows the deidentified patient number. BAL fluid antioxidant proteins (**c**, peroxiredoxin 1, PRX1; **d**, peroxiredoxin 6, PRX6; **e**, mitochondrial glutathione reductase, GR; **f**, thioredoxin 1, Trx1; **g**, glutaredoxin 1, Grx1; **h**, catalase, CAT) were significantly different among the patient groups as determined by pairwise t-test. The data are the mean ± SD and statistically different comparisons are indicated as * *p* ≤ 0.05, ** *p* ≤ 0.007, *** *p* ≤ 0.0005.

To interrogate the consistency of molecular features and pathways associated with the observed groupings in Fig. 1, the proteomic and redox proteomic data were further analyzed by ANOVA with Fisher’s least significant difference (LSD) post-hoc test. In the plasma data, no proteins passed the stringent significance threshold (*p* < 0.05 and false discovery rate (FDR) < 10%), although potentially important trends centered around the complement and coagulation components (data not shown). BAL fluid data provided a more robust differentiation among patient groups, with 328 proteins and 30 reversibly oxidized proteins driving patient group assignment. The top 25 most significant BAL fluid proteins, ranked by raw p-value and passing the 10% FDR threshold, are presented as heatmaps in Fig. 3a and b, with detailed results in Supplemental Tables S1a (proteomics) and S1b (redox proteomics).

In the BAL fluid proteomic data (Fig. 3a), most proteins exhibited markedly lower levels in Group A relative to Groups B and C. Several of these proteins are associated with inflammatory responses and dysregulated energy metabolism and align with the classification of Group A as having the highest disease severity. Among the most significant were vacuolar protein sorting-associated protein 35 (VPS35; P_FDR_ = 1.62E-5), ubiquitin-like modifier-activating enzyme 1 (UBA1; P_FDR_ = 1.54E-5), and key enzymes involved in glucose metabolism, including pyruvate kinase (PKM; P_FDR_ = 3.74E-4), isocitrate dehydrogenase (IDH1; P_FDR_ = 3.1E-4), L-lactate dehydrogenase subunit A and B (e.g., LDHB; P_FDR_ = 1.54E-5), and alpha-enolase (ENO1; P_FDR_ = 3.76E-4).

Redox posttranslational modification of proteins is a well-established mechanism for regulating protein activity and is particularly significant in inflammatory diseases characterized by disrupted mitochondrial function and redox metabolism. In contrast to the proteomic profile (Fig. 3a), where the top 25 most differential proteins generally exhibited greater abundance in Groups C and B compared to Group A, the redox proteomic data revealed distinct subsets of oxidized proteins. One subset was enriched in Group C, while a smaller subset was enriched in Group A (Fig. 3b). The potential implications for oxidation status of the proteins differing by patient Groups are shown in Supplemental Table S1c. The oxidation state of proteins clearly has the potential to contribute to the molecular underpinnings of ARDS as denoted proteins impinge on inflammation, coagulation and vascular systems.

As antioxidant proteins are key regulators of ROS and protein oxidation, the abundance of key antioxidant and redox metabolism proteins was extracted from the BAL fluid proteomic data and determined to be significantly lower in Group A compared to both Groups B and C. These included peroxiredoxin 1 (Fig. 3c; PRDX1), peroxiredoxin 6 (Fig. 3d; PRDX6), mitochondrial glutathione reductase (Fig. 3e; GR), thioredoxin (Fig. 3f; Trx1), glutaredoxin (Fig. 3g; Grx1), and catalase (Fig. 3h; CAT). In addition, the abundance of PRDX6 and catalase were also significantly higher in Group C compared to Group B. The expression profile of other antioxidant proteins across the three patient groups is presented in Supplemental Figure S4a-h and a summary of their relative contribution to the differential antioxidant profile of Group C vs. A is shown using Pareto analysis (Supplemental Figure S4i). Overall, the increased levels of antioxidant proteins in Group C may explain the lower ARDS severity in this group as ROS are well characterized major drivers of the inflammatory response.

### Interrogation of proteomic patterns by classification modeling and pathway analysis

**Fig. 4:**
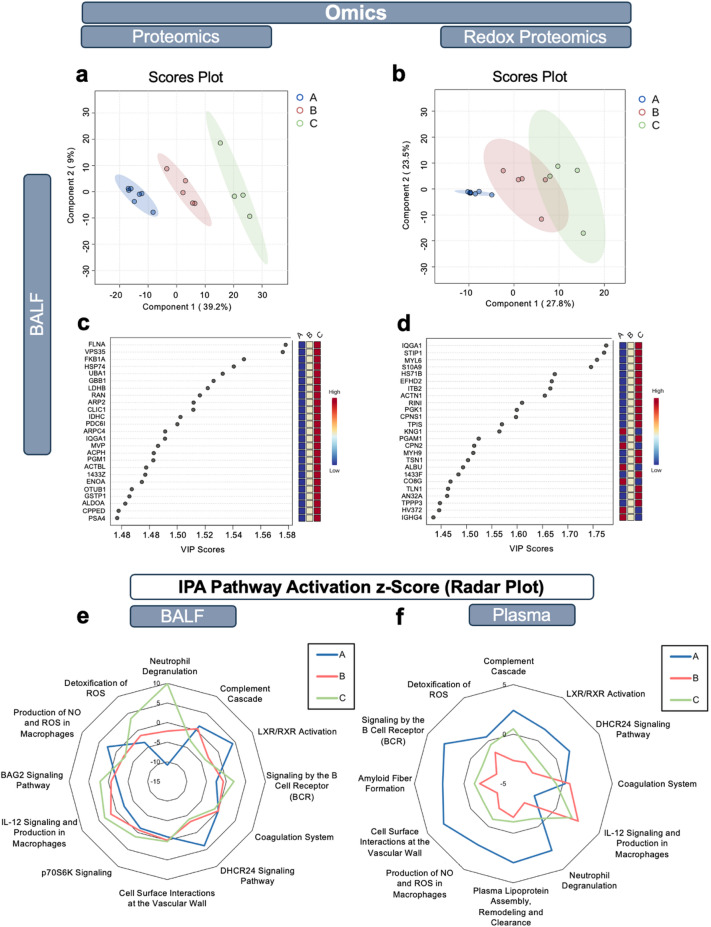
Modeling to identify key proteins drivers and pathways for BAL fluid data. PLS-DA analyses of BAL fluid proteomic (**a**) and redox proteomic (**b**) data show clear patient grouping: groups A (blue), B (red) and C (green). A ranking of the most important proteins (by VIP score) required to classify patients as belonging to Group A, B, or C in BAL fluid proteomic (**c**) and redox proteomic (**d**) data using Day 0 datasets. BAL fluid proteomic signatures were analyzed by Ingenuity Pathway Analysis (IPA) software to identify differences in pathway activation (by Z-score) for proteomic BAL fluid (**e**) and plasma **(f**) data. Pathways were excluded when they described the same event (e.g., complement system vs. complement cascade) and have large protein overlap or when the IPA was unable to estimate a z-score.

Partial Least-Squares Discriminant Analysis (PLS-DA) was applied to the BAL fluid proteomic and redox proteomic data to further investigate the underlying mechanisms of the disease. The fidelity of the BAL fluid proteomics-based patient grouping (Fig. 1e) was consistent with this analysis for both types of data (Fig. 4a-b). A comparable analysis of the plasma proteomic data (Supplemental Fig. S5) revealed a greater overlap among the groups suggesting a loss of local molecular distinguishing features at systemic levels.

To identify the most significant proteins driving the BAL fluid-informed patient groups, the Variable Importance in the Projection (VIP) score was calculated, highlighting the top 25 proteins driving the discrimination between the groups (Fig. 4c-d). As anticipated, the analysis of BAL fluid proteomic data (Fig. 4c) revealed substantial overlap between these proteins and those identified as significantly different by ANOVA among patient groups (Fig. 3a; Supplemental Table S1a). Similarly, the subsets of proteins exhibiting higher oxidation in Group A vs. Group C, and vice versa (Fig. 4d), were consistent with the findings from the ANOVA analysis (Fig. 3b; Supplemental Table S1b).

Ingenuity Pathway Analysis (IPA) was then applied to the BAL fluid and plasma proteomic data to identify the top pathways representing each patient group and the differential pathways activity between the groups using the IPA z-score (predicted activation or deactivation due to several proteins of the same pathway contributing towards activation/deactivation). Since the impact of oxidation on the activity of many proteins involved in the pathways is often unknown, the z-score analysis was applied only to the proteomic data. Similar pathways were significantly detected in both BAL fluid (Fig. 4e) and plasma (Fig. 4f) proteomic data. The pathways identified included those involved in inflammation, redox, and lipid metabolism. Noteworthy pathways were: LXR/RXR activation and DHCR24 signaling, complement cascade, signaling by B cell receptor, coagulation system, cell surface interaction at the vascular wall, IL-12 signaling and production in macrophages, production of NO and ROS in macrophages, detoxification of ROS, and neutrophil degranulation. A unique pathway, amyloid fiber formation, was detected only in the plasma proteomic data and was significant (-log *p* = 12.4; Fig. 4f). This pathway may reflect impairments in the autophagosome and was predicted to be most activated in Group A. A key finding from the BAL fluid data is that almost all overlapping pathways showed mixed activation/deactivation among patient Groups. In contrast, the plasma data showed that pathway activation was a consistent feature for Group A, but not for the other Groups. An exception was IL-12 signaling and production in macrophages, which was suppressed in Group A in both BAL fluid and plasma, consistent with LXR/RXR activation.

## Discussion

Acute respiratory distress syndrome (ARDS) is a clinically and biologically heterogeneous condition, with wide interpatient variability in disease trajectory and response to supportive or anti-inflammatory therapies. In this study, we have started to investigate whether longitudinal bronchoalveolar lavage (BAL) fluid and plasma proteomics and redox proteomics could enable molecular stratification of ARDS patients and reveal mechanistic differences underlying disease severity.

Redox proteomics was selected as a complementary and new approach because, despite extensive evidence linking reactive oxygen species (ROS) to inflammation, fibrosis, and hypoxemia in ARDS, reversible protein oxidation has not been systematically explored in this context. Consistent with this rationale, our data revealed marked dysregulation of redox metabolism and signaling enzymes that distinguished molecular subphenotypes of ARDS. However, given the modest cohort size and exploratory design, these pathway-level findings should be considered hypothesis-generating and will require validation in larger, independent cohorts.

*Compartment-specific proteomic signatures in ARDS.* While both BAL fluid and plasma proteomic profiles were relatively stable within individuals across time points, there were significant differences in the nature of the proteins identified between these biological compartments. BAL fluid data primarily reflected local pulmonary processes, including alveolar macrophage activation, epithelial injury, and localized oxidative stress, whereas plasma captured systemic inflammatory responses and contributions from circulating immune cells such as neutrophils. Notably, pathways related to neutrophil degranulation and systemic inflammation showed contrasting activation patterns between BAL fluid and plasma, underscoring compartment-specific immune regulation in ARDS. Plasma proteomic profiles are also more likely to be influenced by early ICU interventions, including corticosteroids, vasopressors, antibiotics, and sedatives, which may contribute to their more complex hierarchical structure. Together, these observations highlight the value of BAL fluid for interrogating lung-specific molecular pathology and mechanistic drivers of ARDS.

*Identification of molecular subphenotypes.* Using unsupervised hierarchical clustering in conjunction with ANOVA and partial least squares–discriminant analysis (PLS-DA), we identified three recurring molecular subphenotypes (Groups A, B, and C) in this cohort of patients. Although patient grouping showed only weak correlation with APACHE scores, Group A, however, exhibited both the highest APACHE scores and molecular perturbations consistent with higher disease severity. Redox proteomics largely mirrored these groupings, although select patients in Groups B and C (patients 5 and 6) displayed divergent redox profiles, prompting the need for further investigation in larger cohorts. Some patients (e.g., patients 2, 3, and 4) demonstrated remarkably consistent molecular profiles regardless of the specimen (BAL fluid or plasma), the type of analysis (proteomics or redox proteomics), or sampling time despite variable clinical parameters (Table 1; plasma lactate 5.8, 1.3 and 2.9 mmoles/L for patients 2, 3, and 4, respectively), whereas others showed compartment- or time-dependent clustering, reflecting the dynamic interplay between local lung injury and systemic responses.

Analysis of temporal stability revealed less than 20% drift in proteomic profiles over the ICU hospitalization period (up to six days), with most patients clustering consistently across time points (e.g., patients 10 and 13). A subset of patients initially classified as Group A (D0 data) transitioned over the time course of ICU hospitalization toward profiles resembling Groups B or C (D0), potentially reflecting treatment response, a hypothesis that warrants prospective validation.

*Metabolic and inflammatory differences across subphenotypes.* Recent genomic, transcriptomic, and proteomic studies have identified hyper- and hypoinflammatory subtypes of ARDS that differ in immune activation, metabolism, and clinical outcomes^11–14^. Statistical interrogation of the data in our study highlighted molecular features distinguishing Group A from Groups B and C consistent with these prior reports. Group A was characterized by suppressed glycolysis (lower ENO1, PKM, and LDHB) and disruption of the tricarboxylic acid (TCA) cycle (lower IDH1). Reduced IDH1 has been associated with TCA cycle arrest and diversion of citrate toward itaconate production, a metabolite known to suppress inflammation via Nrf2 activation^15,16^. Consistent with this mechanism, Nrf2-mediated oxidative stress responses were deactivated in Group A, and elevated ROS-related markers aligned with greater disease severity and a hyperinflammatory phenotype in agreement with prior ARDS studies^12,14^. Importantly, the integration of redox proteomics further extended these findings by revealing regulatory post-translational modifications particularly associated with hyper- and hypo-inflammatory states through crosstalk with ROS signaling that are not captured by conventional proteomics^17–19^.

In contrast, Groups B and C exhibited proteomic signatures consistent with higher energy metabolism and increased antioxidant capacity, including elevated abundance of ROS-detoxifying enzymes. These differences may reflect younger age or better baseline health in these patients, enabling a more robust metabolic and immune response. Prior BAL fluid proteomic studies^20^ reporting increased compensatory response to injury and stress in surviving ARDS patients further support the relevance of our findings in ARDS pathophysiology and underscore heterogeneity within the syndrome.

*Role of LXR/RXR and DHCR24 signaling.* Several key pathways converged on lipid metabolism and inflammatory regulation, particularly the LXR/RXR axis^21^ and its upstream regulator DHCR24^22,23^. In both BAL fluid and plasma, Group A showed predicted activation of DHCR24 signaling accompanied by LXR/RXR activation, consistent with a hypo-inflammatory state^24^. Activation of LXR/RXR is known to suppress IL-12 signaling^25^, and indeed IL-12 was predicted to be deactivated in Group A. In addition to increased nitric oxide and ROS production, Group A exhibited decreased ROS detoxification, aligning with the greater illness severity in these patients.

LXR/RXR activation also promotes lipogenesis, supplying fatty acids to support mitochondrial oxidative phosphorylation during the hypo-inflammatory phase. The activity of this pathway is regulated by desmosterol, a DHCR24 substrate that acts as a selective LXR agonist^26^. Decreased DHCR24 signaling in Groups B and C may therefore reflect reduced inflammatory state, potentially via suppression of NLRP3 inflammasome activation^27^. These observations raise the possibility that therapeutic modulation of LXR/RXR signaling or DHCR24 activity, for example using LXR agonists or DHCR24 inhibitors such as SH42^26^, could be beneficial in select ARDS subphenotypes, particularly those resembling Group A.

Additional pathway differences included contrasting activation of neutrophil degranulation between BAL fluid and plasma, with strongest deactivation in Group A and activation in Group C within BAL fluid, but opposite patterns in plasma. This suggests greater systemic neutrophil involvement and possible multi-organ injury in Group A. These results are consistent with prior serum proteomics^14^ and tracheal aspirate transcriptomics^12^ findings.

*Novel molecular associations and limitations.* Several proteins not previously implicated in lung injury emerged as potentially relevant. While UBA1 has not been linked directly to ARDS, mutations in this gene drive VEXAS syndrome, a severe inflammatory disorder^28^. Similarly, VPS35, a regulator of kidney nutrient transporters^29^, has been reported to decrease in lung injury models^30^. Both proteins were lower in Group A supporting a broader role in organ injury and stress responses.

A key limitation of this study is the absence of a formal healthy or disease control group. In future studies we intend to include comparator cohorts such as mechanically ventilated ICU patients without ARDS, patients with pneumonia not meeting ARDS criteria, or individuals undergoing bronchoscopy for non-inflammatory indications. Nonetheless, direct comparison across ARDS patients enabled identification of meaningful molecular differences and disease heterogeneity.

*Conclusions.* In summary, this exploratory study provides proof of concept for molecular classification of ARDS using integrated proteomics and redox proteomics, with BAL fluid emerging as a particularly informative specimen for mechanistic interrogation. Despite the limited cohort size, we identified reproducible molecular subphenotypes linked to metabolic state, inflammatory signaling, and clinical severity, with relative temporal stability during ICU hospitalization. Notably, this is the first study to apply redox proteomics to ARDS, revealing reversible protein oxidation as a potentially actionable layer of regulation. With further studies and validation, this approach could support development of a biomarker panel to stratify ARDS patients, guide development of novel therapies, and ultimately improve outcomes.

## Materials and methods

### Patients selection

#### Study approval

This human study was approved by the Wake Forest Baptist Medical Center Institution Review Board (IRB #BG03-081). All research was performed in accordance with relevant guidelines/regulations. Patients, or their authorized representative, gave written informed consent prior to their participation in the study.

#### Sex as a biological variable

Both male (11) and female (5) patients were enrolled in the study. Due to the size of the study, sex was not considered as an independent biological variable in downsteam analysis.

Mechanically ventilated adults (*n* = 16) meeting the Berlin definition of ARDS^31^ were eligible for enrollment. Authorized representatives provided written informed consent. Exclusion criteria included hematologic malignancy, recent chemotherapy, HIV, pregnancy, incarceration, international normalized ratio (a measure of blood clotting rate; INR) > 3, therapeutic oral anticoagulation, platelets < 50,000, acute ischemic heart disease or critical cardiac dysrhythmias, endotracheal tube size < 7.0 mm internal diameter, refractory hypotension, known or suspected elevation of intracranial pressure, or fraction of inspired oxygen (FiO_2_) greater than 90%.

BAL fluid and blood collections were performed within 72 h of ARDS diagnosis (day 0) and up to two additional time points (on days 2–4 and 6–8 post-admission) were collected while the participants remained on mechanical ventilation.

### Collection and processing of specimens

#### BAL procedure

Patients were first pre-oxygenated with 100% oxygen for at least 5 min prior to the procedure and remained on this FiO_2_ for the duration of the procedure. BAL was performed by passing a bronchoscope through the endotracheal or tracheostomy tube and advancing to a wedged position in a segmental or subsegmental bronchus. Segment selection was at the discretion of the research team. However, preference was given to the anterior upper lobe segments, right middle lobe, and lingula since this was the optimal location for both a supine patient and increased BAL fluid return yields. After the bronchoscope was wedged in the desired segment, aliquots of 50–60 mL of sterile 0.9% sodium chloride were instilled in each segment with retrieval performed via gentle manual suction until a total return of at least 30 mL was achieved from each segment, with a maximum instillation of 180 mL per segment.

#### BAL fluid processing

Standard procedures for processing BAL fluid were followed^32^. Briefly, recovered BAL fluid aliquots were pooled in a sterile specimen container and immediately placed on ice. Any visible mucous fluid was removed by aspiration using an 18-gauge blunt needle and discarded. The BAL fluid sample was then centrifuged at 4 °C at 300xg for 10 min. The resulting supernatant was collected, aliquoted and stored at -80 °C until analysis.

#### Blood processing

Blood was collected in 6 mL EDTA-containing vacutainers. The tube was gently inverted 8–10 times to mix before centrifugation at room temperature for 10 min at 1000xg. The plasma was aliquoted and stored at -80 °C until analysis.

### Proteomic and redox proteomic analysis of BAL fluid and plasma specimens

#### Protein extraction

Both BAL fluid and plasma specimens were retrieved from storage in 2020 and processed for mass spectrometry analysis together to prevent batch artifacts.

BAL fluid (250 µL) was supplemented with 10 mM MSTP (4-(5-Methanesulfonyl-[1,2,3,4]-tetrazol-1-yl)-phenol; Xoder Technologies) to block thiols and prevent artifactual protein oxidation during sample processing^33–35^. After a 30 min incubation on ice, the protein fraction was precipitated by addition of ice-cold acetone (4X volume) and overnight incubation at -20 °C. Plasma specimens (10 µL) were first processed to remove the top abundant proteins (e.g., albumin, immunoglobulins) using the Pierce™ Top 2 Abundant Protein Depletion Spin Columns (Thermo Scientific, Cat. No. 85162). The flowthrough was mixed with 10 mM MSTP and incubated on ice for 30 min, followed by the addition of ice-cold acetone (4X volume) and further incubated overnight at -20 °C to precipitate the protein fraction. Next day, the specimens were centrifuged at 16,000 g at 4 °C for 5 min to isolate the BAL fluid and plasma protein pellets for further processing using proteomic and redox proteomic workflows as previously reported^35^.

#### Sample processing for proteomics and redox proteomics

For global proteomics, the protein pellets were suspended in 200 µL of 50 mM NH_4_HCO_3_ containing 10 mM DTT and incubated for 30 min at 56 °C to reduce reversibly oxidized modifications at protein cysteine residues. The newly reduced thiols were alkylated with 30 mM iodoacetamide (IAM) for 30 min at room temperature, protected from light, followed by proteolysis (trypsin: protein = 1:20; Pierce Trypsin Protease, MS Grade, Thermo Scientific) at 37 °C overnight. Next day, the reaction was quenched with 1% formic acid (FA); the peptides were dried using a SpeedVac, resuspended for desalting using C18 tips (Thermo Scientific, Cat. No. 87784), dried again using a SpeedVac, and stored at − 80 °C in preparation for liquid chromatography-mass spectrometry (LC-MS/MS) analysis. For the redox proteomics, after the DTT reduction step, the samples were passed through Bio-Gel P6 spin columns to remove excess DTT and then loaded on columns packed with thiopropyl Sepharose resin (GE Healthcare, Cat. No. 17042001) to bind proteins carrying newly reduced cysteine thiols. The resin was freshly prepared and preconditioned with the binding buffer (50 mM HEPES and 1 mM EDTA, pH 7.5) following the manufacturer’s protocol. The columns were capped and rotated at 4 °C overnight to facilitate covalent binding of proteins to the beads. Next day, the resin was washed, sequentially, with each of the following solutions to disrupt non-covalent protein-protein interactions and reduce non-specific binding of proteins to beads: 8 M urea; 2 M NaCl; 0.1% SDS in PBS; 80% (vol/vol) acetonitrile (ACN) and 0.1% (v/v) trifluoroacetic acid (TFA) in H_2_O; and 50 mM NH_4_HCO_3_.

The proteins covalently bound to the thiopropyl Sepharose resin were subjected to on- beads proteolysis (trypsin:protein = 1:20) at 37 °C overnight in 200 µL 50 mM NH_4_HCO_3_, which was added to each capped column. After digestion, the resin was sequentially washed with the 8 M urea, 2 M NaCl, 80% ACN and 0.1% TFA, and 50 mM NH_4_HCO_3_solutions to remove non-covalently bound peptides. Covalently bound cysteine-containing peptides were eluted with 100 µL elution buffer (25 mM NH_4_HCO_3_ with 20 mM DTT) after incubation for 30 min at room temperature and mixing at 850 rpm. An additional wash was performed with 100 µL of 80% ACN/0.1% TFA and the peptide fractions were combined, dried by SpeedVac, and then stored at − 80 °C in preparation for LC-MS/MS analysis.

#### LC-MS/MS analysis and database search

The resulting peptides for both BAL fluid and plasma global proteomics and redox proteomics were analyzed on a LC-MS/MS system consisting of an Orbitrap Velos Pro Mass Spectrometer (Thermo Scientific, Waltham, MA) and a Dionex Ultimate-3000 nano-UPLC system (Thermo Scientific, Waltham, MA) equipped with an Acclaim PepMap 100 (C18, 5 μm, 100 Å, 100 μm x 2 cm) trap column and an Acclaim PepMap RSLC (C18, 2 μm, 100 Å, 75 μm x 50 cm) analytical column. Peptides were separated during two hours of linear gradient elution, where two mobile phases, water, 0.1% formic acid (A), and 80% acetonitrile, 0.1% formic acid (B) were used. MS spectra were acquired by data-dependent scans consisting of MS/MS scans of the ten most intense ions from the full MS scan with a dynamic exclusion option, which was 30 s. To identify proteins, spectra were searched against the UniProt protein FASTA database *(Homo sapiens*, 20,258 annotated entries, Feb 2018) using Sequest HT algorithm within the Proteome Discoverer v2.2 (Thermo Scientific, Waltham, MA) and the following parameters: FT-trap instrument; parent mass error tolerance of 10 ppm; fragment mass error tolerance of 0.6 Da (monoisotopic); maximum missed cleavage of two sites using the digestion enzyme trypsin, which cleaves specifically at the C-terminal side of Lys and Arg amino acid residues; variable modifications of 16 Da (oxidation) on methionine, 160 Da (MSTP) or 57 Da (IAM) on cysteine. A reversed database search with target decoy PSM validator node (maximum delta Cn 0.05; 0.01 and 0.05 for strict and relaxed target FDR, respectively) was employed for validation. Relative quantitative analysis was performed using the label-free quantification workflow, which considers the total peak areas of identified peptides for each protein normalized to the total ion current.

### Data analysis

Keratin family proteins were excluded from the proteomic and redox proteomic datasets as they were considered potential contaminants^36^. The metadata table generated from LC-MS/MS uploaded on Web-based tool MetaboAnalyst 6 for further data processing, statistical analysis, functional interpretation, and results visualization^37^. The zero or missing variables were replaced with a half of the minimum positive value in the dataset. These values were further normalized to adjust for systematic differences among samples, transformed individual value and adjusted negative values using the square root of the log scale. Supervised Partial Least Squares Discriminant Analysis (PLS-DA) multivariate analysis was performed to interrogate multivariate structure in the data, and the Variable Importance in Projection (VIP) scores were determined to identify the most influential proteins contributing to molecular differences between groups. Unsupervised Ward clustering and Euclidean distancing were used for all hierarchical and heatmap analyses. Pathway analysis was conducted using Ingenuity Pathway Analysis (IPA) software (QIAGEN Inc). For pathway analysis, peak abundances for each protein were averaged across patients and then the averaged levels of each protein in each patient group were compared to the average across all patients to evaluate if the protein was increased or decreased relative to the other patient groups. Relative protein levels were sorted across different pathways and activation or suppression of pathway was determined by Z-score (positive value for activation and negative for suppression). Pathways were ranked by an estimated p-value and the top 12 non-redundant and most significant pathways (*p* < 0.001) were reported and visualized. Other statistical analyses were performed with GraphPad Prism 9 Version 9.3.1 (GraphPad Software, LLC) using data for individual proteins extracted from the proteomic data. Hierarchical clustering analyses were performed in R (version 4.5.2) using RStudio (version 2025.09.2, Build 418). R was used to examine clustering patterns across datasets, assign patient group membership based on dendrogram structure, and compute average silhouette widths to assess overall grouping structure. Statistical methods utilized for each analysis are described in the respective figure legends.

## Supplementary Information

Below is the link to the electronic supplementary material.

Supplementary Material 1

## Data Availability

Proteomic and redox proteomic data generated from this study and used for analyses have been made publicly available. The mass spectrometry data have been deposited to the ProteomeXchange Consortium via the PRIDE partner repository with the dataset identifier PXD060437 and 10.6019/PXD060437.
