# Supplementary material for: A proteomics and redox proteomics approach to understanding ARDS heterogeneity
Source: Sci Rep. 2026 Jan 23;16:6034. doi: 10.1038/s41598-026-35606-2 (PMC12901994; doi:10.1038/s41598-026-35606-2)

Supplemental Materials

A Proteomics and Redox Proteomics Approach to Understanding ARDS Heterogeneity

Thomas E. Forshaw[1,a], Kirtikar Shukla[1], Hanzhi Wu[1.b], Susan Sergeant[2], Jingyun Lee[1], Allen W. Tsang[1], Peter E. Morris[3.c], Kevin W. Gibbs[3], D. Clark Files[3], Cristina M. Furdui[1,,*]

[1] Department of Internal Medicine, Section on Molecular Medicine, Wake Forest University School of Medicine, Winston-Salem, NC, USA

[2] Department of Biochemistry, Wake Forest University School of Medicine, Winston-Salem, NC, USA

[3] Department of Internal Medicine, Section of Pulmonary, Critical Care, Allergy, and Immunologic Diseases, Wake Forest University School of Medicine, Winston-Salem, NC, USA

Current address:

[a] Department of Chemistry, North Carolina Agricultural and Technical State University, Greensboro, NC, USA.

[b] PPD, Middleton, WI, USA

[c] Department of Medicine, Division of Pulmonary/Allergy/Critical Care, University of Alabama at Birmingham, Birmingham, AL, USA

*Corresponding Author: Cristina M. Furdui, Internal Medicine, Section on Molecular Medicine, Wake Forest University School of Medicine, Winston-Salem, NC 27157, USA 336-716-2697, cristina.furdui@advocatehealth.org

***Supplemental Figure S1*. Venn diagrams summarizing the omics data.** The figure shows the number of common and unique proteins quantified based on omics analysis type (proteomics vs redox proteomics, left panels) and biospecimen type (plasma vs BAL fluid, right panels).

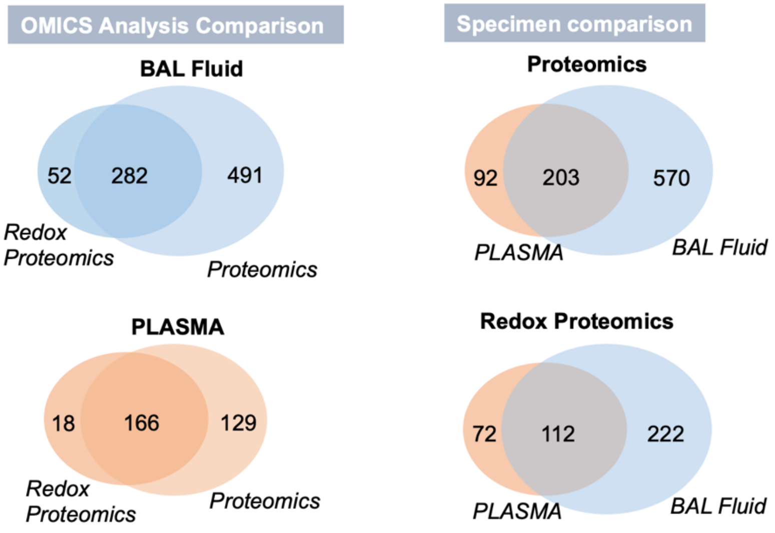

Supplemental Figure S2. **Day 0 (Admission) dendrograms of BAL fluid and plasma Data.** Datasets were analyzed in R-Studio (version 2025.09.2, Build 418) for silhouette-based clustering assessment, and MetaboAnalyst (v6.0) by Ward clustering and Euclidean distancing. Silhouette analysis (**a**) showed average silhouette widths for k=2-4 across BAL fluid and plasmid proteomic and redox proteomic datasets. Given the abundance of proteins (n=773) identified in the global BAL fluid (**b**) compared to the other datasets, this was anticipated to be the most informative for defining the proteomics-informed patient Groups (A, blue; B, red; C, green). This assumption held true for the Group alignments in the BAL fluid redox proteomics (**c**): Group A, 7 of 7 patients clustering together; Group B, 3 of 5 patients clustering together; and Group C, 3 of 4 patients clustering together. The fidelity of the plasma (**d, e**) clustering tended to be lower.

a

Dataset	k=2	k=3	k=4
BAL fluid proteomics	0.335	0.299	0.211
BAL fluid redox proteomics	0.417	0.321	0.323
Plasma proteomics	0.283	0.138	0.109
Plasma redox proteomics	0.422	0.152	0.15

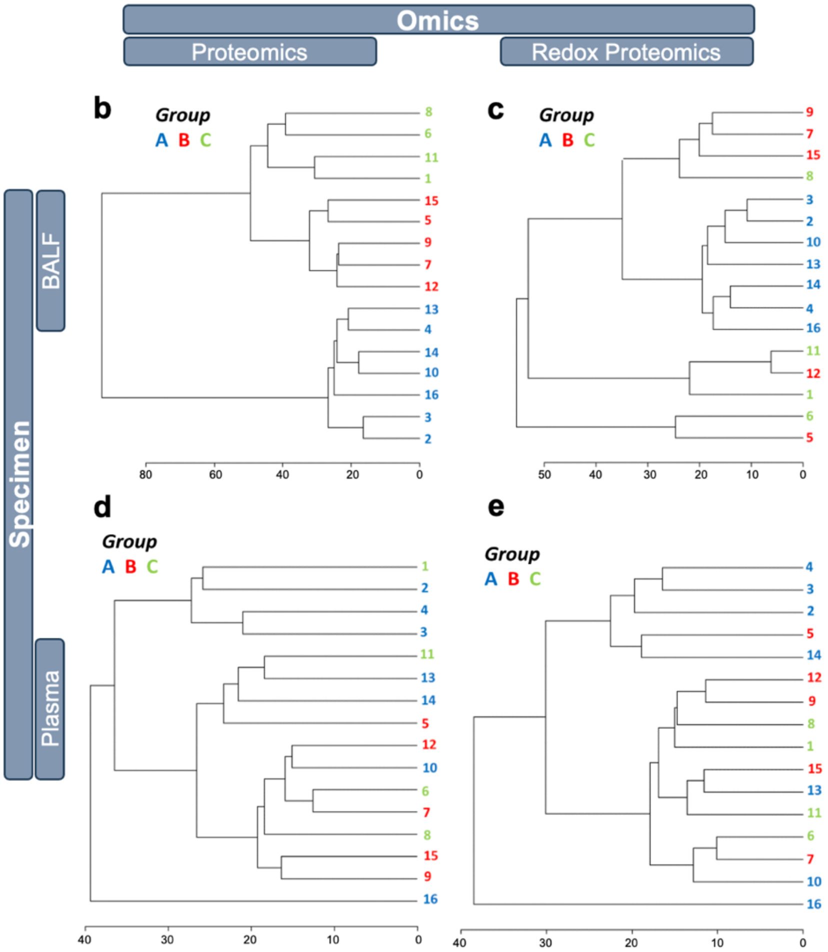

Supplemental Figure S3. **Clinical data across patient groups and other supporting data.** Routine clinical parameters (**a-g**) were stratified by the proteomic-informed patient Groups for comparison by ANOVA or pairwise T-tests. No statistically significant differences were observed for the indicated clinical parameters. The LDH abundance data (**h, i**) were derived from the BAL fluid proteomic data and differed significantly by patient Group, as indicated by the stated ANOVA P-values. The data shown are the mean ± SD.

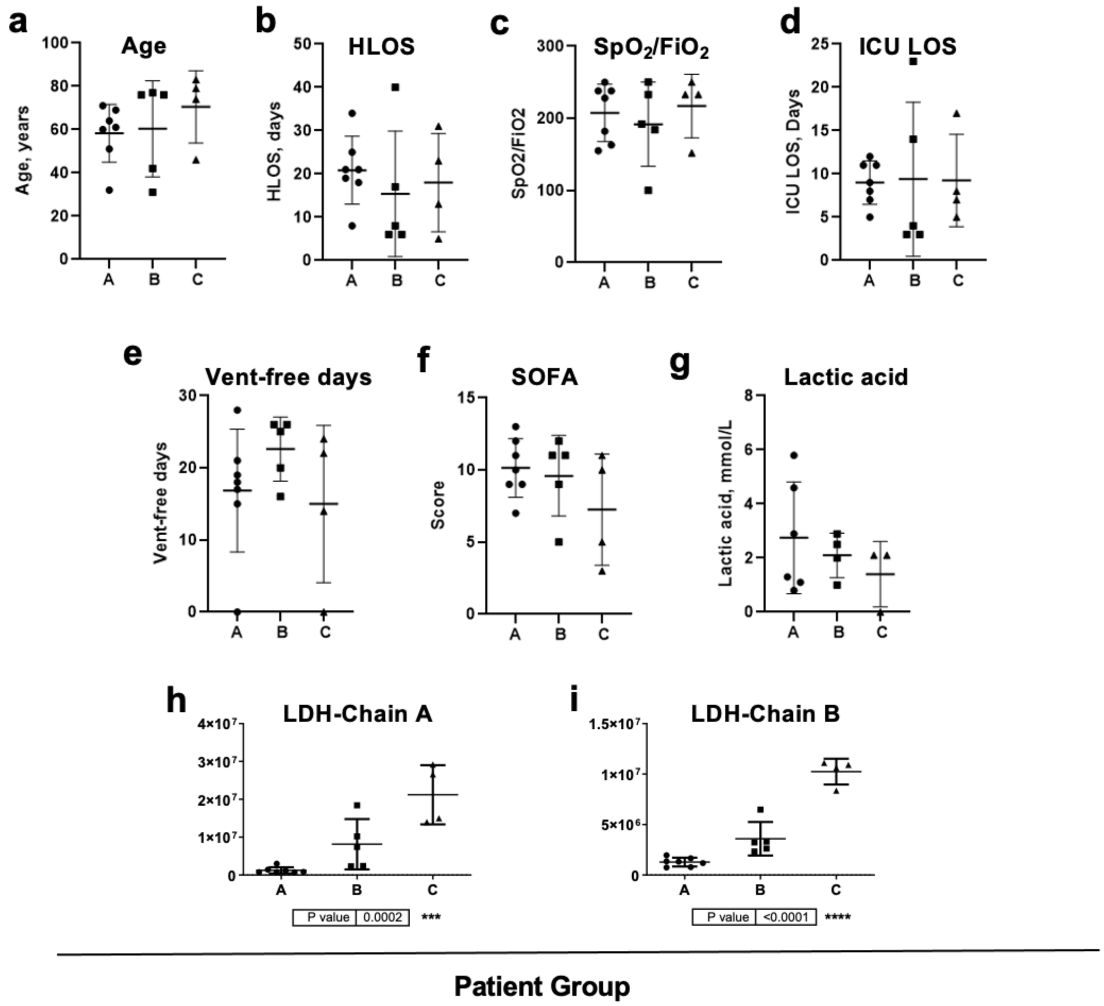

Supplemental Table S1a. **BAL Fluid proteomics Top 25 by ANOVA.** Data are derived from analyses in MetaboAnalyst (v6.0). UniProt protein names are included to facilitate reference to heatmap designations. The table lists proteins in the decreasing f.value.

Uniprot ID	Protein name	f.value	p.value	$-\log_{10}(p)$	FDR	Fisher's LSD
**UBA1**	Ubiquitin-like modifier-activating enzyme 1	79.232	5.23E-08	7.2815	1.54E-05	B - A; C - A; C - B
**LDHB**	L-lactate dehydrogenase B chain	78.469	5.54E-08	7.2563	1.54E-05	B - A; C - A; C - B
**FLNA**	Filamin-A	73.84	7.98E-08	7.0981	1.54E-05	B - A; C - A; C - B
**VPS35**	Vacuolar protein sorting-associated protein 35	69.745	1.12E-07	6.9504	1.62E-05	B - A; C - A; C - B
**FKB1A**	Peptidyl-prolyl cis-trans isomerase A	56.059	4.06E-07	6.3919	4.50E-05	B - A; C - A; C - B
**GBB1**	Guanine nucleotide-binding protein G(I)/G(S)/G(T) subunit beta-1	54.739	4.66E-07	6.3317	4.50E-05	B - A; C - A; C - B
**MVP**	Major vault protein	50.216	7.67E-07	6.1152	6.34E-05	B - A; C - A; C - B
**PSB4**	Proteasome subunit alpha type-4	47.801	1.02E-06	5.9923	7.37E-05	C - A; C - B
**HSP74**	Heat shock 70 kDa protein 4	45.471	1.35E-06	5.8685	8.71E-05	B - A; C - A; C - B
**CLIC1**	Chloride intracellular channel protein 1	39.892	2.83E-06	5.5479	0.000164	B - A; C - A; C - B
**PSA3**	Proteasome subunit alpha type-3	38.72	3.34E-06	5.4757	0.000176	C - A; C - B
**RAN**	GTP-binding nuclear protein Ran	35.925	5.06E-06	5.2956	0.000244	B - A; C - A; C - B
**ARP2**	Actin-related protein 2	35.112	5.74E-06	5.241	0.000256	B - A; C - A; C - B
**1433Z**	14-3-3 protein zeta/delta	34.051	6.79E-06	5.168	0.000281	B - A; C - A; C - B
**ACTBL**	Beta-actin-like protein 2	32.941	8.13E-06	5.0897	0.00031	B - A; C - A; C - B
**IDHC**	Isocitrate dehydrogenase [NADP] cytoplasmic	32.129	9.31E-06	5.031	0.00031	B - A; C - A; C - B
**PDC6I**	Programmed cell death 6-interacting protein	32	9.52E-06	5.0216	0.00031	B - A; C - A; C - B
**PPIB**	Peptidyl-prolyl cis-trans isomerase B	31.703	1.00E-05	4.9997	0.00031	C - A; C - B
**V9HW88**	Calreticulin	31.601	1.02E-05	4.9922	0.00031	C - A; C - B
**ARPC4**	Actin-related protein 2/3 complex subunit 4	30.676	1.19E-05	4.9228	0.000346	B - A; C - A; C - B
**KPYM**	Pyruvate kinase PKM	29.957	1.36E-05	4.8677	0.000374	B - A; C - A
**ENOA**	Alpha-enolase	29.564	1.46E-05	4.837	0.000376	B - A; C - A; C - B
**IQGA1**	Ras GTPase-activating-like protein IQGAP1	29.423	1.49E-05	4.8259	0.000376	B - A; C - A; C - B
**AATC**	Aspartate aminotransferase, cytoplasmic	28.39	1.80E-05	4.7436	0.000427	C - A; C - B
**GDIR1**	Rho GDP-dissociation inhibitor 1	28.277	1.84E-05	4.7344	0.000427	B - A; C - A

Supplemental Table S1b. ***BAL fluid* redox proteomics Top 25 by ANOVA.** Data are derived from analyses in MetaboAnalyst (v6.0). UniProt protein names are included to facilitate reference to heatmap designations. The table lists proteins in the decreasing f.value.

Uniprot ID	Protein name	f.value	p.value	$-\log_{10}(p)$	FDR	Fisher's LSD
**STIP1**	Stress-induced-phosphoprotein 1	45.562	1.34E-06	5.8734	0.000401	C - A; C - B
**IQGA1**	Ras GTPase-activating-like protein IQGAP1	23.077	5.28E-05	4.2772	0.007652	B - A; C - A; C - B
**MYL6**	Myosin light polypeptide 6	21.437	7.65E-05	4.1162	0.007652	B - A; C - A; C - B
**S10A9**	Protein S100-A9	19.791	0.000114	3.9448	0.008516	B - A; C - A; C - B
**ITB2**	Integrin beta-2	15.7	0.000341	3.4673	0.019008	B - A; C - A
**EFHD2**	EF-hand domain-containing protein D2	15.233	0.000391	3.4073	0.019008	B - A; C - A; C - B
**HS71B**	Heat shock 70 kDa protein 1B	14.819	0.000444	3.3531	0.019008	B - A; C - A
**ACTN1**	Alpha-actinin-1	13.455	0.000682	3.1664	0.025563	B - A; C - A; C - B
**TPPP3**	Tubulin polymerization-promoting protein family member 3	12.139	0.001062	2.9739	0.0354	C - A; C - B
**KNG1**	Kininogen-1	11.547	0.00131	2.8827	0.036014	A - B; A - C
**RINI**	Ribonuclease inhibitor	11.525	0.001321	2.8793	0.036014	B - A; C - A; C - B
**CPNS1**	Calpain small subunit 1	11.135	0.001523	2.8174	0.036242	B - A; C - A; C - B
**PGK1**	Phosphoglycerate kinase 1	11.051	0.001571	2.804	0.036242	B - A; C - A
**TPIS**	Triosephosphate isomerase	10.602	0.001858	2.7309	0.039817	B - A; C - A
**ALBU**	Albumin	9.5504	0.002807	2.5517	0.056146	A - B; A - C
**CPN2**	Carboxypeptidase N subunit 2	8.9592	0.003583	2.4458	0.065091	A - B; A - C
**PGAM1**	Phosphoglycerate mutase 1	8.8902	0.003689	2.4331	0.065091	B - A; C - A
**IGHG4**	Immunoglobulin heavy constant gamma 4	8.5096	0.004341	2.3625	0.069451	A - B; A - C
**MYH9**	Myosin-9	8.4789	0.004399	2.3567	0.069451	C - A
**TSN1**	Tetraspanin-1	8.1737	0.005029	2.2986	0.072068	B - A; C - A
**1433F**	14-3-3 protein eta	8.0711	0.005263	2.2788	0.072068	C - A; C - B
**FHR2**	Complement factor H-related protein 2	8.0618	0.005285	2.277	0.072068	A - B; A - C
**CO8G**	Complement component C8 gamma chain	7.8195	0.005894	2.2296	0.076878	A - B; A - C
**TLN1**	Talin-1	7.4121	0.00711	2.1481	0.087584	B - A; C - A
**A1BG**	Alpha-1B-glycoprotein	7.2758	0.00758	2.1203	0.087584	A - B; A - C

Supplemental Table S1c. ***Oxidized BAL fluid proteins significantly different by patient groups that potentially contribute to ARDS.*** BAL fluid redox proteomics that were significantly enriched Group C or Group A were explored to evaluate relevance to ARDS. Data are derived from analyses in MetaboAnalyst (v6.0). The FDR-adjusted p values are given.

		BALF, P_{FDR}	Effect when Oxidized	Reference
Enriched Oxidation in Group C				
RAS GTPase activating-like protein	IQGAP1	7.65E-03	regulates endothelial migration	(1)
phosphoglycerate mutase 1	PGM1	0.065	unknown in ARDS	
			upregulation in the neutrophils of patients with sepsis	(2)
Stress-induced-phosphoprotein 1	STIP1	4.01E-04	Anti-inflammatory in mouse spinal injury model; modulates NFκB activity	(2, 3)
Protein S100-A9	S100-A9	0.0085	anti-inflammatory	(4)
phosphoglycerate kinase 1	PGK1	0.036	decreased enzyme activity, which inhibits cellular production of IL1β and IL6	(5)
			(when reduced, activation improved survival in mouse model of sepsis)	(6)
Enriched Oxidation in Group A				
kininogen 1		0.036	oxidation decreases activity	(7, 8)
			decreased activity contributes to impaired coagulation and vasodilation	(9)
albumin		0.056	associated with a poorer prognosis	(10)
complement component C8		0.076	unknown in ARDS	

References for Supplemental Table S1c

1. Kaplan N, Urao N, Furuta E, Kim SJ, Razvi M, Nakamura Y, et al. Localized cysteine sulfenic acid formation by vascular endothelial growth factor: role in endothelial cell migration and angiogenesis. Free Radic Res. 2011;45(10):1124-35.
2. Sehgal R, Kaur N, Maiwall R, Ramakrishna G, Maras JS, Trehanpati N. Plasma Proteomic Analysis Identified Proteins Associated with Faulty Neutrophils Functionality in Decompensated Cirrhosis Patients with Sepsis. Cells. 2022;11(11).
3. Jin H, Ge X, Huan Z, Yao H, Xu C, Cai J. Stress-induced phosphoprotein 1 restrains spinal cord ischaemia-reperfusion injury by modulating NF-kappaB signalling. J Cell Mol Med. 2021;25(24):11075-84.
4. Lim SY, Raftery MJ, Goyette J, Hsu K, Geczy CL. Oxidative modifications of S100 proteins: functional regulation by redox. J Leukoc Biol. 2009;86(3):577-87.
5. Dekany K, Vas M. Inactivation of pig muscle 3-phosphoglycerate kinase by thiol modification depends on reagent size. Eur J Biochem. 1984;139(1):125-30.
6. Chen X, Zhao C, Li X, Wang T, Li Y, Cao C, et al. Terazosin activates Pgk1 and Hsp90 to promote stress resistance. Nat Chem Biol. 2015;11(1):19-25.
7. Kozik A, Golda A, Mak P, Suder P, Silberring J, Barbasz A, et al. Myeloperoxidase-catalyzed oxidative inactivation of human kininogens: the impairment of kinin-precursor and prekallikrein-binding functions. Biol Chem. 2011;392(3):263-74.
8. Nieziolek M, Kot M, Pyka K, Mak P, Kozik A. Properties of chemically oxidized kininogens. Acta Biochim Pol. 2003;50(3):753-63.
9. Duchene J, Ahluwalia A. The kinin B(1) receptor and inflammation: new therapeutic target for cardiovascular disease. Curr Opin Pharmacol. 2009;9(2):125-31.
10. Bonifazi M, Meessen J, Perez A, Vasques F, Busana M, Vassalli F, et al. Albumin Oxidation Status in Sepsis Patients Treated With Albumin or Crystalloids. Front Physiol. 2021;12:682877.

Supplemental Figure S4. **Comparison analysis of additional antioxidant proteins across groups.** BAL fluid antioxidant proteins (**a-h**) were evaluated (one-way ANOVA) across the patient Groups and found to be significantly different as indicated: * p=<0.05, ** p=<0. 002. The data are the mean ± SD. The Pareto plot (**i**) shows the relative contribution of the respective antioxidant protein based on fold change (FC) for the proteomic-informed Group C vs Group A (C/A; based on mean values).

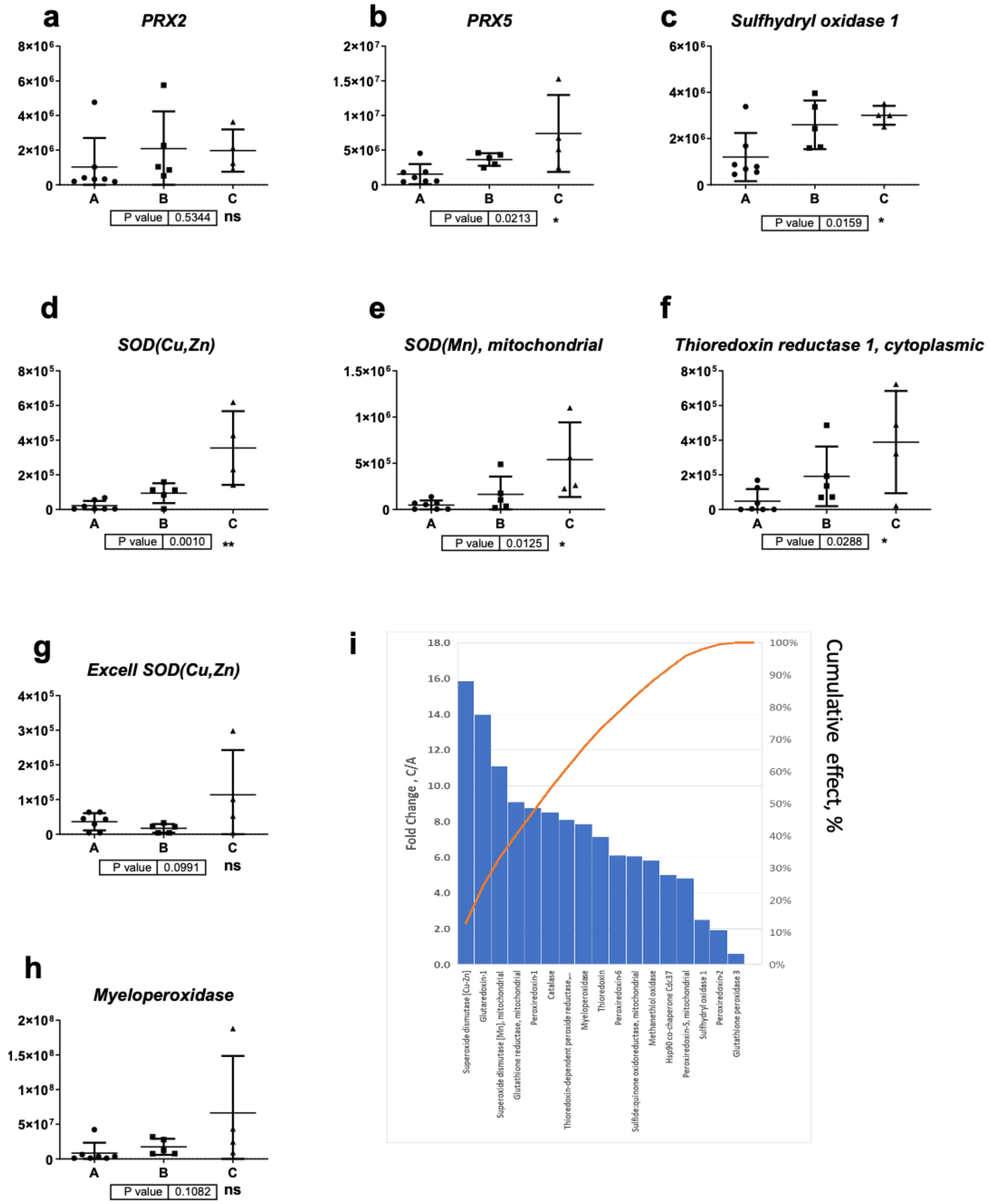

Supplemental Figure S5. **Plasma proteomics PLS-DA analysis.** Plasma proteomic data (study admission/Day 0) was subjected to PLS-DA analysis using MetaboAnalyst, v6.0. The resultant Scores plot showed considerable overlap among the patient Groups (ovals represent the 95% confidence interval). Pairwise analysis by PERMANOVA (in MetaboAnalyst) yielded p values of Groups A vs B (0.655), Groups A vs C (0.45) and Groups B vs C (0.214).

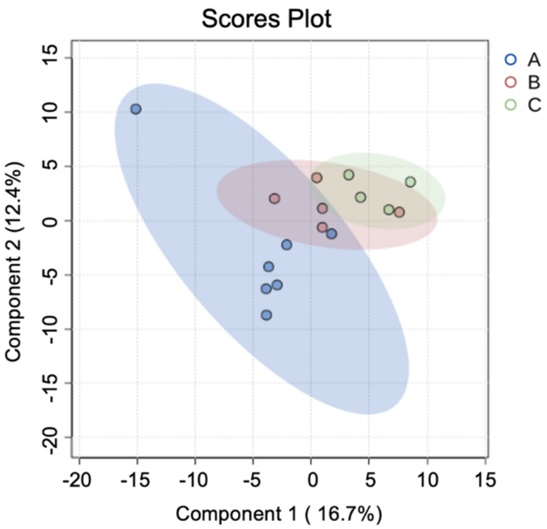

Supplement: Supplementary file 1 — Supplementary Material 1 [file 41598_2026_35606_MOESM1_ESM.pdf]
